# Profile and outcome of post stroke patients managed at selected public primary care health centres in Peninsular Malaysia: A retrospective observational study

**DOI:** 10.1038/s41598-018-36154-0

**Published:** 2018-12-19

**Authors:** Aznida Firzah Abdul Aziz, Mohd Fairuz Ali, Mohammad Fhaisol Yusof, Zuraidah Che’ Man, Saperi Sulong, Syed Mohamed Aljunid

**Affiliations:** 1 Department of Family Medicine, Faculty of Medicine, Universiti Kebangsaan Malaysia Medical Centre, Kuala Lumpur, Malaysia; 2 Department of Medicine, Hospital Tawau, Ministry of Health Malaysia, Tawau, Sabah, Malaysia; 3 Research Support Unit, Department of Emergency Medicine, Hospital Canselor Tuanku Muhriz, Universiti Kebangsaan Malaysia Medical Centre, Kuala Lumpur, Malaysia; 4 Centre for Clinical Epidemiology and Biostatistics, Hunter Medical Research Institute, The University of Newcastle, Callaghan, New South Wales, Australia; 5 Department of Community Health, Faculty of Medicine, Universiti Kebangsaan Malaysia Medical Centre, Kuala Lumpur, Malaysia; 6 International Centre for Casemix and Clinical Coding, Faculty of Medicine, Universiti Kebangsaan Malaysia Medical Centre, Kuala Lumpur, Malaysia; 7 Department of Health Policy and Management, Faculty of Public Health, Kuwait University, Hawally, Kuwait

## Abstract

Data on post stroke outcomes in developing countries are scarce due to uncoordinated healthcare delivery systems. In Malaysia, the national stroke clinical practice guideline does not address transfer of care and longer term post stroke care beyond tertiary care. Hence, post stroke care delivery may be delivered at either tertiary or primary care facilities. This study aimed at describing patients’ characteristics and outcomes of post stroke care delivered by the primary care teams at public primary care healthcentres across Peninsular Malaysia. Multi staged sampling was done to select public primary care health centres to recruit post stroke patients. At each health centre, convenience sampling was done to recruit adult patients (≥18 years) who received post stroke care between July-December 2012. Baseline measurements were recorded at recruitment and retrospective medical record review was done simultaneously, for details on medical and / or rehabilitation treatment at health centre. Changes in the measurements for post stroke care were compared using paired t-tests and Wilcoxon Rank test where appropriate. Total of 151 patients were recruited from ten public primary care healthcentres. The mean age at stroke presentation was 55.8 ± 9.8 years. Median duration of follow up was 2.3 (IQR 5.1) years. Majority co-resided with a relative (80.8%), and a family member was primary caregiver (75.%). Eleven percent were current smokers. Almost 71.0% of patients achieved BP ≤ 140/90 mmHg. Only 68.9% of the patients had been referred for neurorehabilitation. Percentage of recorded data was highest for blood pressure (88.1%) while lowest was HbA1c (43.0%). For clinical outcomes, systolic and diastolic blood pressure, triglyceride level and calculated GFR (eGFR) showed statistically significant changes during follow up (p < 0.05). Post stroke care at public primary care healthcentres showed benefits in stroke risk factors control (i.e. hypertension and dyslipidaemia) but deterioration in renal function. A more structured coordination is needed to optimise post stroke care beyond acute phase management for patients who reside at home in the community.

## Introduction

Stroke is a disease of acute onset with long standing evolution of complications^1^. In low and middle income countries, access to specialised stroke care is fragmented and patchy, concentrated mostly at urban areas^2,3^. However, even in countries with the best of public healthcare facilities, the fragmentation of post stroke care is well-acknowledged in literature^4–9^.

In Malaysia, patients who receive treatment for acute stroke in tertiary or secondary hospitals, are usually managed at hospital-based specialist outpatient or primary care clinics after discharge^10^. Patients in the acute stage are admitted on average between 6.4 days to 9.8 days^8,9,11^ before being discharged, mostly to be cared in their homes by family members. Patients, who do not receive treatment at hospitals during initial acute stroke period, may also seek treatment from the primary care team at public healthcentres or private general practitioners^12^. To date, there is no national stroke incidence or prevalence. Furthermore, there is no protocol which specifically addresses the transfer of care from tertiary to primary care for long term post stroke management at community level. Patients or their caregivers have the option to either be followed up at public or private primary care health facilities of their choice. The primary care team then takes on the role of navigating the care coordination across multidisciplinary teams, mostly when and if required^13^. Stroke survivors have a pooled cumulative risk of 11.1% at 1 year, 26.4% at 5 years and 39.2% at 10 years after the first stroke^14^. Hence the need to ensure that secondary preventive measures are in place in order to reduce recurrent stroke and major cardiovascular events. The role of the primary care team in providing secondary preventive care for post stroke or completed stroke patients has been increasingly acknowledged in the past decade^10,15–17^, especially in providing supportive care for patients who have either been discharged from hospitals or reside in underserved areas where specialist stroke services are lacking or geographically challenged^18,19^.

In Malaysia, Family Medicine Specialists (FMS) are entrusted to lead the primary care services at the public healthcentres. Apart from providing long term monitoring for stroke risk factor(s) modification, which essentially consists of management of non-communicable diseases, the coordination of neurorehabilitation is challenging. The lack of knowledge and awareness on post stroke rehabilitation services are identified as causes which prevent delivery of comprehensive post stroke care for patients residing at home in the community^12,20,21^.

In terms of post stroke outcome studies, the data is usually homogenous and highlights stroke outcomes at specific periods of stroke recovery^22^. Most studies focus on stroke outcomes in the acute clinical phase, as recovery mostly takes place within the first six months after a stroke^23,24^. However, the longer term issues which need to be better coordinated are prevention of recurrent strokes^22^, addressing physical disabilities and psychological and cognitive problems which extend beyond acute period^25^ and community reintegration^26^. In a setting where transfer of care is largely uncoordinated and fragmented across different care environments (i.e. secondary healthcare facilities, public vs private healthcare institutions) this situation is further compounded by absence of community based-post stroke registry to assess effectiveness of long term care provision for this group. Local data on clinical outcomes of patients who received treatment at primary care i.e. after their discharge from hospital (or diagnosis) is scarce. To the time this study was conducted, there was no published information regarding post stroke outcomes beyond discharge from tertiary care, in particular those who were discharged to public primary healthcare facilities for long term monitoring. In this study we look at the baseline demographic and rehabilitation profiles of post stroke patients receiving care at public primary care health centres across Peninsular Malaysia and their clinical outcomes achieved. It is hoped that the information from this study will guide improvements in service provision for post stroke patients receiving treatment at public primary care health centres.

## Methods

This study employed retrospective observational methods, assessing the outcomes of post stroke patients attending public primary care healthcentres, from their first visit up to the time of the study recruitment. This study was conducted between 1^st^ July 2012 and ended on 31^st^ December 2012. Multistage sampling methods were employed.

### Selection of study sites

Several public primary care health centres were randomly chosen from the individual state health websites, to represent the northern, southern, western, central and eastern zones of Peninsular Malaysia. The health centres in East Malaysia were excluded due to lack of funding and logistic difficulties. The family medicine specialists (FMS) in the identified zones were contacted by the researcher via telephone, to obtain current information regarding health centre population demographics, support services provided and the availability of a post stroke registry at the health centres. Snowballing technique was used to identify the other health centres which may have population of post stroke patients in the NCD or Elderly Clinic services.

Total of sixteen FMS’ working in six different states in Peninsular Malaysia were invited to take part in this trial. Study sites were selected if the primary care service is a public healthcare facility, had an active registry of patients attending NCD or elderly clinic service based at the health centre. Centres with low burden of post stroke patients were excluded from the study.

Altogether, ten^10^ FMS’ from total of ten primary care health centres decided to participate in the trial. The remaining six^6^ FMS’ who refused participation cited reasons such as low estimated numbers of post stroke patients at the health centre or the FMS and staff at the health centre could not commit to the trial protocol. All the FMS’ were given three months before the trial commenced, to identify and list all post stroke patients receiving treatment at the respective health centres. This was deemed necessary as a specific stroke registry was not available, and manual search had to be conducted among patients who were being treated for non-communicable diseases (NCD). The post stroke patients were under follow up for chronic care i.e. monitoring of stroke risk factor(s), and any other complications related to stroke or new complaints. There was no specific or additional program for post stroke monitoring aside from ensuring treatment targets met the local clinical practice guidelines^27–29^. Patients were seen between 1–4 monthly intervals, depending on their clinical condition.

Inclusion criteria into the study were patients aged 18 years and above and clinically diagnosed with stroke due to any cause by the treating physician, with or without radiological confirmation. These patients must have completed acute stroke treatment, discharged from hospital and referred for long term stroke care at community health centres. Patients who were diagnosed with Transient Ischaemic Attack (TIA), or presented with isolated nerve palsy were excluded from the study.

### Recruitment of subjects

Patients were recruited for the study during their follow-up appointment at the healthcentres during the study period. Consent for participation was obtained from patients and their caregivers. It was considered necessary to obtain informed consent from caregivers as most of the patients were dependant on their caregivers for transportation to the health centres.

Data collected were sociodemographic factors, details of stroke, risk factor(s) for stroke (i.e. blood pressure, lipid profile, glycaemic status, renal function) as well as information on rehabilitation (i.e. functional status and history of rehabilitation done). Review of the patients’ medical records was done simultaneously to gather information or details of management when the patient was first transferred to or referred to the primary care health centres for long term management. Details on neurorehabilitation were obtained from either patient and/or caregiver and rehabilitation records. All the information was checked and verified by the researcher during the monitoring site visits.

### Data Analysis

The quantitative data were analysed using Statistical Package for Social Sciences® (IBM-SPSS) version 21.0 (New York, 2012). Student’s t-Test was used to evaluate statistical significance changes between the mean score of the clinical variables at first visit to the health centre and at trial recruitment. Wilcoxon Rank test was used for non-parametric data. Significant level was set at p-value < 0.05.

This study obtained approval from the Ethics & Innovation Committee, Faculty of Medicine, Universiti Kebangsaan Malaysia (Research ID GUP-UKM-2011-321) as well as Ministry of Health, Malaysia (Research ID: NMRR-11-1074-10358).

### Ethics Approval and Consent to Participate

The Research Ethics Committee of Universiti Kebangsaan Malaysia Medical Centre (Approval reference no: 1.5.3.5/244/UKM-GUP-2011-327), National Institutes of Health Malaysia, Medical Ethics and Research Committee (MREC), Ministry of Health, Malaysia approved this study as well as the informed consent procedure (in 2012). Informed written consent to participate in this study was obtained from all participants.

## Results

A total of 151 patients were recruited from ten public primary care health centres during the study period. The mean age of the participants at the time of recruitment was 60.2 ± 9.5 years. The overall mean age at stroke presentation was 55.8 ± 9.8 years (males 55.8 ± 9.6, females 55.9 ± 10.9). The time to initial contact with a primary care physician after discharge from tertiary care was about 8 weeks (median 57.0 days, IQR 272.0 days), with median duration of follow up at the health centres was 2.3 years (IQR 5.1). The average number of stroke episodes per patient was 1.2 ± 0.5 episodes.

Only 68.9% (104/151) of the patients were referred for formal stroke rehabilitation after diagnosis. The median duration for patients undergoing different rehabilitation for stroke based on type are physiotherapy 6.0 (IQR 9) months, occupational therapy 6.0 (IQR 11.0) months and speech and language therapy 11.9 (SD 9.1) months. Tables 1 and 2 summarises baseline characteristic and clinical profiles of post stroke patients at recruitment into the study. Changes in relevant stroke clinical parameters at baseline recruitment from the first consultation in the primary care health centres are presented in Table 3. There were statistically significant changes for blood pressure, renal function and triglyceride (TG) levels. The reductions in mean blood pressure readings for systolic and diastolic were 8.98 (CI:12.79,4.49) mmHg and 5.20 (CI:7.41,2.98) mmHg respectively. There was slight deterioration in renal function with an eGFR difference of 6.21 (9.63,2.78) mL/min/1.73 m^2^.

**Table 1:** Baseline characteristics of post stroke patients receiving treatment at selected public health centres in Peninsular Malaysia. (N = 151).

Variables	n (%)
Age category	
≤50 years	19 (12.6)
51–60 years	53 (35.1)
61–70 years	48 (31.8)
71–80 years	14 (9.3)
≥81 years	17 (11.2)
Gender	
Male	81 (53.6)
Female	70 (46.4)
Ethnicity	
Malay	80 (53.0)
Chinese	54 (35.8)
Indian	15 (9.9)
Others	2 (1.3)
Education level	
Secondary school	83 (55.0)
Primary school	49 (32.5)
Did not attend school	12 (7.9)
College/university	7 (4.6)
Occupational status	
Unemployed/retiree	90 (59.6)
Housewife	28 (18.5)
Fixed Salaried employee	23 (15.2)
Self-employed	10 (6.6)
Living Arrangements	
With spouse	74 (49.0)
Staying with married children	33 (21.9)
Staying with unmarried children	15 (9.9)
Alone	10 (6.6)
Nursing home	2 (1.3)
Others	17 (11.3)

**Table 2:** Clinical profiles of stroke survivors receiving treatment at selected public primary care health centres in Peninsular Malaysia. (N = 151).

Variables	
Age at stroke, Mean (SD)	
Male	55.8 ± 9.6
Female	55.9 ± 10.9
Duration of stroke	
≤2 years	75 (49.7)
>2 years	76 (50.3)
Stroke subtypes	
Ischaemic	107 (70.9)
Unspecified	30 (19.8)
Haemorrhagic	14 (9.3)
Functional status (Modified Barthel Index scores)	
≥90	98 (65)
<90	53 (35)
Neurorehabilitation program post stroke	
Yes	104 (68.9)
No	47 (31.1)
Physiotherapy (after acute stroke period)	
Yes	90 (59.6)
No	61 (40.4)
Occupational Therapy (after acute stroke period)	
Yes	31 (20.5)
No	120 (79.5)
Speech & Language therapy	
Yes	22 (14.6)
No	129 (85.4)
Smoking status	
Non smoker	103 (68.2)
Ex-smoker	31 (20.5)
Current smoker	17 (11.3)
Baseline BP	
≤140/90	109 (72.2)
>140/90	42 (27.8)
Co-morbid conditions/Stroke risk factors	
Hypertension	147 (97.4)
Dyslipidaemia	136 (90.1)
Diabetes mellitus	103 (68.2)
Atrial fibrillation	5 (3.3)

Values are in frequency (%) unless specified.

**Table 3:** Changes In Clinical Parameters Of Post Stroke Survivors In Primary Care Health Centres.

	Baseline At Recruitment		First Consultation		P
Systolic BP^#^ (mmHg)	133.86	(18.73)	142.50	(21.77)	**<0.05**
Diastolic BP^#^ (mmHg)	77.75	(11.21)	82.95	(11.79)	**<0.05**
Total Cholesterol^#^ (mmol/L)	4.88	(1.014)	5.00	(1.02)	0.25
LDL^#^ (mmol/L)	2.98	(0.93)	3.11	(0.99)	0.19
HDL^#^ (mmol/L)	1.16	(0.31)	1.21	(0.32)	0.08
Mean eGFR^# +^ (mL/min/1.73 m^2^)	68.33	(25.99)	74.54	(24.62)	**<0.05**
TG^*^ (mmol/L)	1.34	(0.89)	1.44	1.00	**<0.05**
HbA_1c_* (%)	6.85	2.00	7.00	4.00	0.17

^#^Mean (SD).

^*^Median (IQR).

^+^Calculated glomerular filtration rate using the CKD-EPI Creatinine 2009 formula on www.mdrd.com.

**Figure 1:**
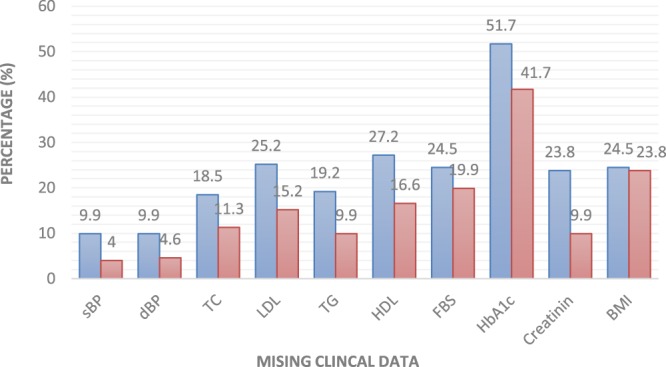
Missing clinical data based on medical case record review of first visit at primary care health centre and during recruitment.

In terms of retrieving the clinical data from the medical case records, the highest percentage of missing data was the HbA_1c_ levels which was not available (first visit 51.7%, recruitment 41.7%) of all the patients recruited. The detail for other missing clinical data is illustrated in Fig. 1.

## Discussion

Our study is a first attempt to document outcomes of post stroke patients managed in the community with follow up in public primary care health centres in Peninsular Malaysia after their discharge from hospitals for the acute stroke episode.

This study focused on the secondary prevention of stroke i.e. risk factors management; in which the majority of the cases were managed by primary care physicians despite still having tertiary care appointments. In most cases, patients are discharged to be fully managed by the primary care team and are only referred back to tertiary hospitals when necessary^12^. This shared care approach with the primary care team was to cater for the long gaps in the follow up appointments with the hospital based Specialist/Neurology clinics. This was normally due to congestion at Specialist/neurologist clinics. For this reason, continuing follow up in the primary care level can be seen as a safety netting measure to monitor post stroke complications and the risk of recurrence stroke was relatively high up to 5 years after the initial stroke^30–33^.

In terms of neuro-rehabilitation after stroke, almost one third of patients (31.1%) were not referred to any rehabilitation facility for assessment or intervention. This could partly be attributed to the lack of awareness among physicians regarding the role of neuro-rehabilitation for stroke patients^21^ or lack of coordination of care transition once the patient is discharged from hospital after the acute episode. This issue partly also stems from the fact that most medical curriculums in this country do not incorporate rehabilitation as a compulsory subject during training of physicians, leaving only those who are interested to pursue knowledge on rehabilitation as an elective subject. A proper assessment by a rehabilitation physician or at least by the various multidisciplinary allied health personnel cannot be overemphasized, as consequences of long term disability and dependency on caregivers are avoidable, in most cases^34–36^.

Nevertheless, the time interval from hospital stroke care to first consultation with the primary health care doctors varies widely. This is due to various factors that may delay the transfer of care to the community health care doctors. Confusion with the multiple clinic appointments, logistic reasons and caregiver-related issues are reasons given for the delay; explaining the wide standard deviation of the timing for first primary care consultation which was up to 252 days, (median 57 days) after discharge from acute care. This finding was almost similar to an earlier work by our group at an urban based primary care clinic in which the first contact with primary care averaged (median) at four months post discharge^10^. As such, this highlighted the need for a standardised protocol to facilitate the transfer of care of patients to community based health care centres, or to incorporate better safety netting measures during the period of transition. This is particularly important for those who are discharged early from tertiary centres after the acute episode, where the average duration of stay in the hospital during the acute phase is less than a week^11^. We also postulate that varied practices of pre-discharge planning in most tertiary centres could have contributed to the study findings. Ideally, pre-discharge planning could aid successful care transitions between tertiary and primary care, with the aim to reduce complications related to stroke^2,37,38^.

This study has highlighted the lack of coordination of post stroke care beyond the acute stroke phase. Fragmentation and poor coordination of care for post stroke patients occurs even in the most advanced healthcare systems in developed countries^2^. Developing countries however, face tougher challenges in terms of geographical coverage, availability of specialised or dedicated stroke care facilities and services as well as lack of established community social support services for non-institutionalised patients^39^. Another reason for the fragmentation of care is most likely related to the lack of coordination between tertiary and primary care facilities, resulting in significant numbers of stroke patients failing to attend regular follow up and deprived of vital long term continuous management, which explains why only 68.9% of the patients had undergone some form of rehabilitation post stroke. This may also explain the lack of data and evidence on long term outcomes of post stroke patients in this country.

Hence, it is our recommendation that proper transfer of care protocol is addressed, incorporating detailed pre-discharge planning with relevant safety netting advise conveyed to the patient and their caregivers to overcome fragmented care as soon as patients return home. In developing countries where there is limitation in tertiary inpatient rehabilitation services, early discharge with support from facilities available within community settings may be the only workable solution, a compromised version for the recommended early supported discharge model as touted by Fisher and colleagues^40^.

The role of the primary care team in providing continuous coordinated care cannot be overemphasised and should not be limited to simply monitoring of stroke risk factors (i.e. hypertension, dyslipidaemia and diabetes mellitus), but also include addressing stroke related complications such as physical disability, swallowing problems and mental health related issues such as depression and dementia. As the stroke related complications may not be part of the standard care protocols for most chronic care programs conducted at public primary care health centres across the country, having a standardised care protocol or care algorithm may be helpful for the primary care team to use as reference to link them to resources available within the community.

Our study showed that the important modifiable risk factors for stroke such as hypertension (both systolic and diastolic blood pressure), and dyslipidaemia (i.e. triglyceridaemia) management showed significant improvements. Our study covered health centres which were located in the urban, suburban and rural areas of the country. These public primary care health centres were headed by Family Medicine Specialists, who are orientated to the shared care approaches for chronic disease management protocols within the public healthcare system. The findings of this study suggest that community primary care doctors may have a positive impact in optimising some of stroke risk factor(s) control. Therefore, enabling provision of long term stroke care to be extended to areas which lack access to specialist stroke care services is especially relevant where the follow up appointments are spaced between six to nine months apart, due to high number of patients at Specialist stroke care facilities.

However, more can still be done as it is interesting to note that HbA_1c_ levels was among the highest percentage of missing information among post stroke patients who had type 2 diabetes mellitus. Feedback obtained from study sites regarding the lack of HbA_1c_ monitoring results was due to health centre budget restrictions during the study period, whereby testing was extremely limited. Another area which lacked documentation was recording BMI of patients’, with patients deemed physically unfit for weight measurement as a common reason.

It is also alarming to note that patients have deteriorated eGFR during follow up at the health centres over a median period of 2.3 (IQR 5.1) years. Furthermore, the percentage of missing data for recording of Creatinine level was recorded at 23.8% in the patients’ medical records. The declining renal function among the study population may have been on-going prior to stroke, as the majority of patients have multiple co-morbid conditions or stroke risk factors, diagnosed either before or at the time of diagnosis. The decline in GFR as a consequence of polypharmacy is another possibility to reconsider. The effect of cyanocobalamin or vitamin B12 ingestion which has been known to reduce GFR^41^ was considered, however, only one out of the total 151 respondents was prescribed vitamin B12. Wu *et al*.^42^ concluded that the risk of developing chronic kidney disease (CKD) was higher in patients with stroke (aHR1.43, 95% CI: 1.36–1.50, p < 0.001), and more so when patients had concomitant diabetes mellitus (aHR 2.12, p < 0.001), hyperlipidaemia (aHR1.53, p < 0.001) and gout (aHR1.84, p < 0.11)^42^. In addition, the risk of progression to CKD and ESRD was significantly higher among stroke patients, regardless of age, gender, co-morbidities or long term medications^32^. Hence, it is imperative that measures to improve not only documentation as well as monitoring of renal function among stroke patients be done on a regular basis, while limiting factors which may further contribute to deterioration of renal function among post stroke patients.

There are several, unavoidable limitations, which may have influenced the study outcomes. Our study mainly concentrated on patients who were able to attend follow up at primary care health centres and did not include those who were home bound due to severe disability arising from the initial stroke or complications of stroke or caregiver-related issues.

The data gathered was from a small population and therefore, future studies with larger sampling should be conducted to allow generalisation. However, this study did highlight the shortcomings in terms of transfer of longer term stroke care between tertiary and primary care services.

The management of post stroke patients residing at home in the community can be potentially optimized at public primary care healthcenters if the primary care team is guided to provide an integrated care service using available healthcare services within the community. The efficacy of an integrated care plan for delivery of post stroke care services based at primary care should be evaluated and compared to determine its effectiveness with the current care provision in a cost effectiveness evaluation to justify its implementation nationwide. In conclusion, monitoring of post stroke patients at public primary care healthcentres showed possible significant improvements in blood pressure control and triglyceride levels, but with declining eGFR levels. Post stroke monitoring of glycemic control, as well as optimization of rehabilitation services available at community level are areas which can be further improved upon.

## Data Availability

.The datasets generated during and/or analysed during the current study are available from the corresponding author on reasonable request.
